# Expected effects of the US tax reform on other countries: global and local survey evidence

**DOI:** 10.1007/s10797-020-09618-1

**Published:** 2020-08-11

**Authors:** Dorine Boumans, Clemens Fuest, Carla Krolage, Klaus Wohlrabe

**Affiliations:** ifo Institute, Munich, Germany

**Keywords:** US tax reform, Tax Cuts and Jobs Act, Corporate tax, Firm responses, Survey, Germany, H25, H32, D22, F23, E62

## Abstract

The Tax Cuts and Jobs Act constitutes the largest change to the US tax system since the 1980s and thoroughly alters the way in which multinational companies are taxed. Current assessments on the reform’s international impact vary widely. This article sheds light on the tax reform’s expected effects on other countries. We first use representative German business survey data to analyze the impact of the reform on German firms. Many firms with substantial US revenues or capacities in the USA intend to expand US investment in response to the reform, in particular large firms and manufacturing companies. The effects on investment in Germany are ambiguous: While some firms substitute between investment locations, others expand in both countries. We subsequently extend our analysis to a global level using worldwide survey data. The results suggest a negative impact on tax revenues and investment in countries with close economic ties to the USA.

## Introduction

On December 22, 2017, US President Donald Trump signed into law the Tax Cuts and Jobs Act. This reform constitutes the most substantial overhaul of the US tax system since President Reagan’s 1986 reform and changed both the corporate and the personal income tax. Most notably, the reform reduced the statutory federal corporate income tax rate from 35 to 21 percent and thoroughly changed the taxation of multinational firms. In addition to converting from a worldwide tax system with deferral to a modified territorial tax system, the TCJA introduced new international provisions (BEAT, FDII and GILTI) affecting the taxation of multinational income.

With many of the TCJA’s provisions targeting multinationals, the reform does not only have a far-reaching impact in the USA, but around the globe. Beyond the demand stimulus expected from the tax reform, the reform may induce companies to shift investment as well as taxable profits to the USA. However, some provisions may exert countervailing effects and induce investment in other countries. So far, no clear consensus has emerged on the extent of these effects (Kopp et al. 2019). However, when deciding whether and how to design a policy response, the international implications of the TCJA are of utmost importance to policy makers.

Against this background, this paper gathers survey evidence to shed light on the reform’s potential international effects on investment, trade and tax revenues. As the TCJA’s economic effects are largely contingent on firm responses to the reform, this paper mainly relies on representative survey evidence from German firm surveys. As Germany is among the world’s most export intensive economies (Statista 2020) and among the largest providers of US inbound FDI (Jackson 2017), information on German firm responses is instructive for assessing the tax reform’s international effects.

Our most important findings are as follows: While most German firms do not plan to alter their investment, an important share of firms with US exposure, measured by revenue generated in the USA or by having US subsidiaries, plans on increasing US investment. The effects on German domestic investment are ambiguous. While some firms intend to invest more in both countries, others intend to cut investment in Germany and replace it by higher investment in the USA. Companies which intend to invest more in the USA also plan to increase exports to the USA. The idea that companies will invest and produce in the USA to replace exports from Germany finds little support in our data. We subsequently supplement our findings with results from a worldwide economic expert survey to gauge the reform’s effects on a wide array of countries. Our global survey results suggest a negative impact on tax revenues and investment in countries with close economic ties to the USA.^1^

The reminder of the paper is as follows. First, we explain some background of the TCJA and review the existing literature. Then, we introduce our three surveys. Our survey-based results are presented along the various impacts of the TCJA such as the tax burden or investment.

## Institutional background and the international impact of the Tax Cuts and Jobs Act

### Institutional background

Many of the TCJA’s provisions exert an impact on firms around the globe, particularly in the realm of corporate taxation. First, the corporate tax rate cut directly affects after-tax profitability, leaving more cash for investment, salaries or dividends to shareholders. The reform also lowers the tax burden on pass-through entities and temporarily allows immediate expensing of short-lived capital investments. By lowering marginal effective tax rates (METR), at least for equity-financed investments (Gravelle and Marples 2020), the TCJA thereby increases incentives for domestic investment.^2^

Second, the reform thoroughly alters the tax treatment of multinationals by converting to a modified territorial tax system, which exempts dividends from domestic taxation. Prior to the reform, US companies faced taxation on their worldwide income. Taxes paid abroad were credited against US tax. However, the taxation of foreign profits that did not qualify as Subpart F income was deferred until repatriation, i.e., did not need to be paid as long as earnings were kept abroad. For this reason, many firms retained profits in their foreign subsidiaries to avoid the high tax rates upon paying dividends to the US parent. Following the reform, repatriated dividends are exempt from domestic taxation, but a transition tax was levied on past foreign profits. This tax of between 8 and 15.5% was levied irrespective of whether repatriation takes place. Exempting repatriated dividends from taxation means that cash accumulated abroad is now more easily available in the USA, for example for investment purposes, dividends to shareholders or share repurchases. However, while businesses could not use their unrepatriated earnings to engage in transactions that benefit shareholders, they could nevertheless invest these earnings in the US financial market. Some analysts therefore suggest that repatriation does not make much of a difference.^3^ The varying assessments of the effects of repatriation highlight how little is known about the potential effects of the reform.

Third, further provisions aim at curbing tax base erosion. These measures may in some cases even increase the tax burden for US multinationals, and their incentives are not as straightforward (Chalk et al. 2018; Clausing 2020). The “Global Intangible Low Taxed Income” (GILTI) and the “Foreign-Derived Intangible Income” (FDII) provisions were designed to remove tax incentives to shift profits derived from intangible assets to low-tax countries. GILTI effectively constitutes a minimum tax on foreign earnings. While the first ten percent return on assets is tax exempt, a minimum tax of 10.5 percent is levied on earnings exceeding this threshold. This may lead to countervailing effects (Clausing 2019; Dharmapala 2018): For one, the exemption provides an incentive to invest in foreign assets. Also, since the TCJA constitutes a global minimum tax, firms may offset earnings from tax havens with earnings from high-tax countries. For firms with substantial income from tax havens, this may actually incentivize increasing investments in high-tax countries. In contrast, the FDII is a tax deduction for export-oriented US corporations. This tax benefit applies to income that is both attributable to intangibles held in the USA and derived from foreign sales above a normal return on assets. While constituting an incentive to hold intangible assets related to exports in the USA, the FDII also encourages the offshoring of real investment (Clausing 2020; Sanchirico 2018). Finally, the “Base Erosion and Anti-Abuse-Tax” (BEAT) is a minimum tax on US profits intended to limit profit shifting to other countries. This tax is charged on payments to foreign related entities above a threshold. This lowers profit-shifting incentives.

Fourth, the TCJA contains several further provisions that impact business decisions. Among others, the amortization period for R&D expenses will be extended to 5 years from 2022 onwards, again raising the cost of investment. Furthermore, the reform abolished the alternative minimum tax. It also altered loss carry over rules, eliminating loss carry backs and limiting loss carry forwards to 80% of subsequent years’ income.^4^

In addition, the TCJA also entails significant changes for the taxation of personal income. These include rate cuts, an increase in the standard deduction as well as limits on itemized deductions. As opposed to the corporate tax reform, these provisions are temporary and expire after 2025.

While this paper mainly focuses on the international implications of the corporate income tax provisions, our survey data also reflects views about the impact of the entire reform package, including the significant personal income tax cuts. This is the case as firm assessments may also account for changing US consumption patterns driven by the reform.

### Literature overview

A growing number of contributions in the literature discuss the reform’s impact, partly based on macroeconomic simulation models. One strand of the literature focuses on effects on the US economy and suggests avenues for tax policy improvements (e.g., Auerbach 2018; Chalk et al. 2018; Clausing 2020; Gale et al. 2018; Kopp et al. 2019; Slemrod 2018). Other studies specifically target the international impact of the reform (e.g., Clausing 2019; Dharmapala 2018; Gravelle and Marples 2020). Some of these studies use simulation models to quantify international tax spillovers. Focusing on the tax rate cut and calibrating their model with parameters found in the literature, Beer et al. (2018) find declining investment and declining taxable profits of multinational firms reported in other countries. Similarly, Spengel et al. (2018) and Heinemann et al. (2018) assess the effects of the reform on FDI flows between Europe and the US based on the effective tax burden for cross-border investments. They conclude that the effective tax burden both on European FDI in the USA and on US FDI in Europe falls, and additional US inbound investment from the EU rises, while outbound investment in the EU increases at a lower magnitude. Low-tax countries, such as Ireland, are predicted to benefit more than high-tax countries, such as Germany. Focusing explicitly on Germany, Christofzik and Elstner (2018) find a positive impact on German GDP and an increase in the current account using structural vector auto-regressions. However, these simulation studies abstract from many of the TCJA’s international tax provisions. Therefore, actual effects might substantially deviate.

So far, studies using firm-level responses are scarce. For one, Gaertner et al. (2020) assess stock returns around the TCJA’s major tax reform events, finding substantial heterogeneity around the globe. While the majority of foreign firms experienced positive returns, Chinese firms overwhelmingly experienced negative returns. Hanlon et al. (2019) analyze company statements about actions following the TCJA. While they find that 22% of S&P 500 firms announced a positive investment response, responses differ by firm characteristics. Notably, companies with a high ratio of cash taxes to pretax income are more likely to announce additional investment, whereas multinational companies are less likely to announce responses than companies solely based in the USA. Domestic responses have also been covered by US firm surveys (see Kopp et al. 2019, for an overview). According to the NABE quarterly Business Conditions Survey, 11% of firms attributed rising investment to the TCJA in 2018, while 24 percent of small business owners surveyed by the National Federation of Independent Business planned to expand investment with their tax savings. While these findings are broadly consistent with our survey results, the focus of our surveys differs. While other surveys capture US responses to the reform, and thereby have a domestic focus, our surveys shed light on the international implications of the TCJA, focusing on German firms with considerable exposure to the US market.

## Evidence: global and German firm surveys

We assess the impact of the TCJA within the scope of three surveys administered by ifo Institute. While results from the two firm-level surveys help shed light on German firms’ perceived impact of the reform, we subsequently complement these findings with results from a global expert survey. Many studies assess tax reform implications within the scope of macroeconomic models, for example, by using vector autoregressive models (see, e.g., Mertens and Ravn 2013). Firm surveys, on the other hand, have the advantage of measuring the (perceived) impact of tax reforms at the microlevel of economic agents. While administrative tax data are typically only available with a substantial time lag, survey results provide a more readily available picture of firm responses. Experts may have—potentially individually different—models under consideration and other sources of information to assess shocks to an economy. Surveying experts can condense this information.

Both firm-level surveys are representative for German firms. As the design of the surveys differs, they jointly provide a more nuanced and in-depth picture of international firm responses. The first business survey was conducted in March 2018 as a part of the regular monthly ifo business survey. This survey is the basis for the ifo business climate index, which is considered the most important leading indicator for German economic activity.^5^ The ifo business survey is a representative monthly survey of about 9000 German firms.^6^ The four main sectors covered by the survey are industry (about 2500 answers), trade (2200), services (2500) and construction (1800). The regular questionnaire contains monthly, quarterly, biannual and annual questions and is filled in for the most parts by the owner or the CEO of the firm (Sauer and Wohlrabe 2019).^7^ The questions about the US tax reform were included as supplementary questions and were answered by 4231 firms.^8^ The largest share of responses is in the service sector with 38%, closely followed by industry with 36%. The least answers were received from the trade sector. The questions were not posed in the construction sector. As many small- and medium-sized enterprises do not operate on the US market, we show separate results for firms with US exposure. This subgroup consists of 550 companies who derive at least 5% of their revenue from the USA. These firms are primarily found in the industry sector (see Table 1).

**Table 1:** Participation in the two firm surveys

	Panel A: First ifo firm survey March 2018				Panel B: Second ifo firm survey April/May 2018			
	All firms		Firms with at least 5% US revenue		All firms		Firms with US production	
Industry	15,31	36%	375	68%	416	33%	85	45%
Construction	–	–	–	–	111	9%	3	2%
Trade	1095	26%	43	8%	158	13%	14	7%
Services	1605	38%	132	24%	578	46%	87	46%
Total	4231	100%	550	13%	1263	100%	189	15%

The table reports participation rates for the two firm surveys across sectors and US exposure

The second business survey was conducted by ifo Institute for the non-profit organization *Stiftung Familienunternehmen* (Foundation Family Enterprises). This is a joint project where ifo executes an annual representative survey with changing main topics. The 2018 survey addressed international tax competition. Based on a stratified representative sample,^9^ more than 70,000 firms were contacted either via letter or electronic mail. The survey period was between May and June 2018. In the end, 1263 firms filled in a questionnaire, corresponding to a response rate of about 2%. Most answers came from the service sector (46%) followed by industry (33%). The distribution of respondents across sectors and firm size is representative of the German economy as a whole. Only the trade sector is somewhat underrepresented, whereas the construction sector is oversampled (Table 1).^10^

Our firm-level analysis focuses on survey questions which specifically address the US tax reform and its potential impact on German firms. The first ifo business survey (Panel A) focuses on the impact of the US tax reform on the tax burden of German firms, on their investment choices and on trade with the USA. While the second business survey (Panel B) also addresses firms’ tax burden and investment choices, it has a slightly different focus, notably differing with respect to the classification of firms with substantial exposure to the USA. While the first survey uses a firm’s US revenue share to classify US exposure, the second survey directly identifies firms with existing US production sites. The exact wording of the questions in both surveys can be found in Appendix.

To broaden our analysis beyond Germany, we supplement our results with a global survey of economic experts, assessed by the ifo World Economic Survey (WES). Our global-level evidence is based on the views of around 1000 economic experts^11^ from 120 countries who participate in the WES.^12^ In selecting economic experts for the WES panel, emphasis is placed on their professional competence in economic matters and inside knowledge of their countries. This is guaranteed by screening their education and current affiliation.^13^ Each quarter, the panelists are asked to assess main macroeconomic variables in their respective country. In addition, the survey includes supplementary questions about political or economic issues of current interest. In the April 2018 survey, the recent US tax reform was the topic of these additional questions. In total, 1155 experts from 119 countries participated in the April survey in 2018. 907 respondents answered the supplementary questions (see Table 7 for the distribution across world regions).

These unique data sources offer the possibility to analyze the expected impact of the US tax reform on German businesses and to contrast these findings with other countries based on economic expert knowledge. The three surveys allow us to assess the effects along the different parameters such as tax burdens and revenues, investment choices, trade effects, as well as other relevant aspects, such as the location of intellectual property rights.

## Survey results: expected impact of the TCJA

### Overall impact and reactions

To provide an overview of whether firms are affected by the TCJA, we first distinguish between two aspects: whether the TCJA has an impact on firms and whether firms adjust their behavior in response. That is, the first aspect encompasses changes to a firm’s tax burden and, in case of the second survey, also to a firm’s competitive position. These aspects do not necessarily entail any behavioral changes on the part of the firm. In contrast, the second aspect covers whether a firm actively reacts to the reform, e.g., by adjusting investment or production strategies. These different adjustment patterns will subsequently be analyzed in more detail in the following sections. Responses are depicted in Table 2. Note that responses across both surveys are not readily comparable, as questions in both surveys address slightly different aspects of the reform (see questionnaire in Appendix).

**Table 2:** Overall effects of the TCJA on the firm level

	All firms (%)	Firms with at least 5% US revenue (%)
*Panel A: First ifo firm survey March 2018*		
Overall affectedness	8	13
Impact (change in tax burden)	4	16
Reaction	5	8

	All firms (%)	Firms with US production (%)
*Panel B: Second ifo firm survey April/May 2018*		
Overall affectedness	26	85
Impact (change in tax burden and competitive position)	10	40
Reaction	22	73

This table reports various measures of the effect of the TCJA on German firms

According to the first survey, 8% of all firms are affected by the reform. This share increases to 13% among firms with at least 5% US revenue. Across all firms, respectively, about 5% report to be either impacted though changes in their tax burden, or to actively react. Among firms with substantial US revenue, the share of impacted firms is twice as large as the share of firms that plan to actively react.

In the second survey, 26% of all firms report to be affected by the TCJA, either through changes in the tax burden or through changes in their competitive position. This share rises to 85% if we only consider firms with US production sites. For all firms, but also for the firms with US production sites, it is striking that a larger fraction reports to actively react to the changes in US tax law than claims a direct impact. 22% of the surveyed firms plan to react to the tax reform (see Table 2), with details on their responses outlined in later sections.

The difference between the two surveys with respect to affectedness, impact and reaction is notable. First and foremost, this is attributable to the more comprehensive set of questions in the second survey (see Appendix). Second, the two surveys take a different approach to measuring US exposure: Raising substantial revenue is not the same as having a production plant in the USA. As many firms possibly export to the USA without any local production, this might lead to the first survey’s lower perceived affectedness.

**Fig. 1:**
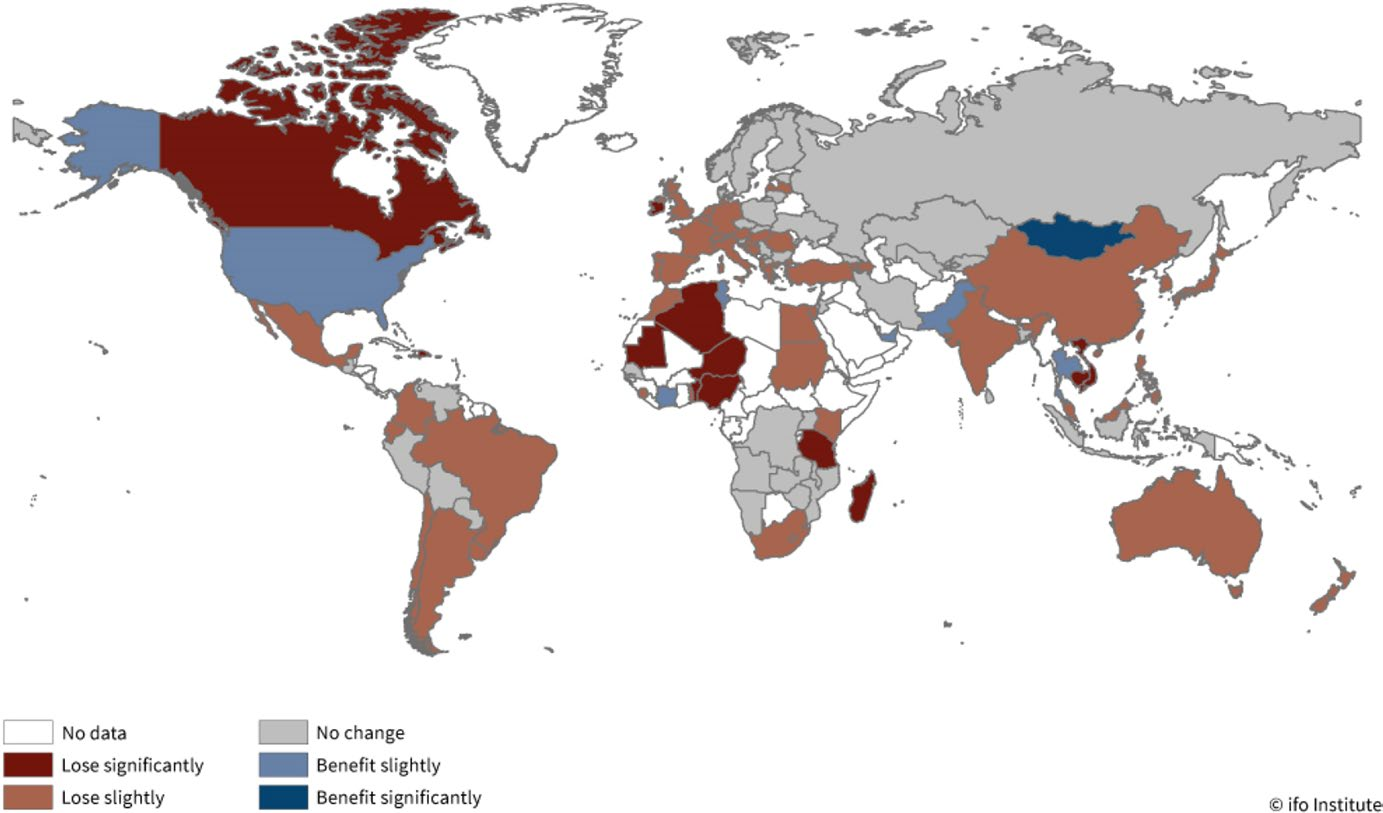
Who stands to lose or benefit from changes in US tax policy? *Note* Data based on the answers of WES II/2018. Colors represent the answer categories after recoding, where lose significantly was coded − 2, lose slightly − 1, no change 0, benefit slightly 1, and benefit significantly 2. Then, an average of the answers was taken where − 2 till − 1 represents lose significantly, − 0.9 till − 0.2 lose slightly, − 0.2 till 0.2 no change, 0.2 till 1 benefit slightly, and 1.1 till 2 benefit significantly

To put these results in a broader light, we asked the WES panelists if they expected either benefits or losses for their own country. This initial assessment underlines the relevance of the reform for the global economy: Fig. 1 shows that experts around the world do expect their country to be affected by the TCJA. Experts in the USA are envisaging a slight benefit from the tax reform, whereas negative assessments are most prevalent in countries with substantial US FDI (Jackson 2017): in Canada, Germany, Ireland, Mexico, Switzerland and the UK. In these countries, three quarters or more of respondents anticipate negative consequences. However, most respondents from the Netherlands, which is one of the largest US FDI destinations, reported not to be affected by the reform (86.7%), while 31.2% expect to lose slightly.^14^

Table 3 addresses the anticipated effects of the reform on the USA and on different worldwide regions.^15^ Responses clearly indicate that especially in regions with close trade ties to the USA, respondents most frequently anticipate negative outcomes (EU15 and other advanced economies). Regions with comparably less economic integration with the USA, for example the Commonwealth of Independent States (CIS) and Eastern Europe, are expected to be less affected. While respondents from the USA lean toward a positive assessment of the reform’s impact, roughly a third of respondents think the USA will be negatively affected by this reform. These perceptions may be driven by several factors, including the impact the reform might have on tax planning structures, tax revenues and investment. To further assess these impacts, the next section explores these in more detail, drawing together evidence from the firm surveys as well as the expert survey.

**Table 3:** Survey results of the expert survey—general impact

	N	Lose significantly	Lose slightly	No change	Benefit slightly	Benefit significantly
USA	36	3%	31%	19%	33%	14%
EU15	292	4%	50%	37%	9%	1%
Newer EU members	126	0%	22%	70%	7%	0%
Other advanced economies	100	5%	55%	23%	15%	2%
CIS and Emerging Europe	87	5%	18%	70%	7%	0%
Emerging Asia	51	6%	44%	40%	9%	0%
Latin America	107	13%	39%	38%	8%	2%
Africa	108	16%	26%	47%	10%	0%
Total	907	56	348	399	92	12

This table reports the answers of the ifo expert survey on the general impact of the TCJA on experts’ respective countries

### Tax burden and revenues

Lowering (or in some cases possibly raising) tax payments is the most immediate channel through which the TCJA may affect firms. Panel A of Table 4 shows the expected impact on the tax burden of firms, also distinguishing short- and long-run effects for the first survey. As expected, only a small fraction of German firms are directly affected by the reform. Unsurprisingly, the share of firms envisaging a changing tax burden is larger in the subgroup of firms with US exposure. In this group, 14% anticipate their tax burden to decline. This number rises in the long run and is also increasing in firm size and with the share of revenues derived from the USA. More substantive long-run responses may also be due to the transition tax on past foreign profits, which could increase tax payments in the short run. In contrast, 8% of all firms with substantial US exposure expect a rising tax burden in the long run. This effect could be due, for instance, to the more restrictive treatment of R&D spending from 2022 onwards, or it could be related to tax avoidance measures such as BEAT and GILTI. The share of firms expecting tax cuts is higher in the second survey (11%) and increases to 59% when considering only firms with US production. Comparably few respondents also claim to be impacted by the changing loss carry over rules (see Table 4 Panel B).

**Table 4:** The TCJA’s effect on firms’ tax burden

	*N*	Decrease (%)	No change (%)	Increase (%)
*Panel A: First ifo firm survey March 2018*				
Short-run				
All firms	4116	3	96	1
Firms with at least 5% US revenue	540	14	82	4
Long-run	4063	3	93	3
All firms	4063	3	93	3
Firms with at least 5% US revenue	531	17	75	8

	*N*	All firms (%)	Firms with US production (%)	
*Panel B: Second ifo firm survey April/May 2018*				
Tax cut	1261	11	59	
Loss deduction rules	1261	2	11	

	*N*	Decrease (%)	No change (%)	Increase (%)
*Panel C: ifo expert survey April 2018*				
USA	42	86	5	10
EU15	269	21	75	4
Newer EU members	124	5	94	1
Other advanced economies	97	31	55	14
CIS and Emerging Europe	85	10	82	7
Emerging Asia	46	19	79	1
Latin America	106	13	85	2
Africa	105	12	85	3

This table reports the perceived effect of the TCJA on firms’ tax burden measured at the firm level and complemented by assessments by experts on the country level

Responses in Panel C of Table 4 show the expected impact on tax revenues across the world, assessed by the respective countries’ experts. A clear majority of US respondents expects decreasing tax revenues. This is in line with the Congressional Budget Office’s (2018) and the Joint Committee on Taxation’s (2017) estimates. Most respondents from other countries, however, do not expect the reform to have a substantial impact on their countries’ revenues. The largest effects are anticipated in non-EU advanced economies, where 31% expect a decrease and 14% anticipate an increase in revenue. Explanations are conceivable for both assessments. If profits or investments are moved toward the USA, other countries’ tax revenues could possibly decrease. However, firms around the world may also benefit from increasing consumption in the USA and may even direct some of their possible revenue increases toward investment in other countries.

### Investment

The TCJA’s impact on investment constitutes one of the most important aspects of the tax reform. On the one hand, numerous provisions, such as the rate cut, make investing in the USA comparatively more attractive. This might lead to investments being shifted from other high-tax countries, such as Germany, to the USA. On the other hand, some provisions may well exert a countervailing effect. Notably, firms with substantial activity in tax havens may face an incentive to invest more in high-tax countries, as earnings from these countries may offset earnings from tax havens under GILTI.

We therefore examine the TCJA’s effect on firms’ planned investment in both the USA and Germany. While a clear majority of firms in the first survey do not plan on altering their investment strategies, Table 5 shows that 14% of the firms with US exposure intend to invest more in the USA. This number rises to 31% among the firms expecting a decline in the tax burden they face, suggesting a strong firm response to tax incentives. As expected, only a few businesses plan to reduce US investment. In a similar spirit, the second firm survey asked whether firms plan to extend existing or build up new investment capacities. 34% of firms with US subsidies intend to expand their existing capacities, while 17% want to invest in new ones. This again indicates a substantial investment response to tax incentives.

**Table 5:** Effects on investment in the USA and in Germany

	*N*	Decrease (%)	No change (%)	Increase (%)
*Panel A: First ifo firm survey March 2018*				
Investment in the US				
All firms	3372	4	92	3
Firms with at least 5% US revenue	492	6	80	14
Firms expecting a reduction in their tax burden	157	8	61	31
Investment in Germany				
All firms	3571	2	92	6
Firms with at least 5% US revenue	489	3	87	10
Firms expecting a reduction in their tax burden	153	10	79	11
Firms planning to increase investment in the US	105	26	61	13

	*N*	All firms (%)	Firms with US production (%)	
*Panel B: Second ifo firm survey April/May 2018*				
Extension of US capacities	1261	5	34	
New investment capacities	1261	3	17	

	*N*	Decrease (%)	No change (%)	Increase (%)
*Panel C: ifo expert survey April 2018*				
USA	41	2	39	59
EU15	266	27	65	8
Newer EU members	125	16	78	6
Other advanced economies	97	41	50	9
CIS and Emerging Europe	86	28	69	4
Emerging Asia	46	38	54	8
Latin America	105	39	54	7
Africa	102	22	62	16

	*N*	Decrease (%)	No change (%)	Increase (%)
*Panel D: ifo expert survey METR classification*				
METR <10	125	33	62	5
METR 10-18.7	156	26	71	3
METR 18.8-25	198	44	50	6
METR 25-34.6	162	28	62	10
METR >34.6	87	28	62	11

This table provides evidence on the effect of the TCJA on how German firms’ investment in the USA and Germany will change (Panels A and B). This is complemented by the assessment by experts (Panels C and D)

Table 5 also summarizes the responses regarding investment in Germany. While most businesses do not plan to adjust their German investment, 10% of the firms with US exposure intend to invest more in Germany. In addition to offsetting GILTI, this may have several further reasons: Expanding economic activity may require inputs produced in Germany, and liquidity effects of US tax cuts may also remove constraints on investment in other countries.^16^ However, for many companies, we also find a substitution effect between investment in Germany and the USA. Among the firms which intend to invest more in the USA, 26% intend to cut back on German investment. These are twice as many as those who plan to invest more in both countries. Overall, while investment effects are positive in the USA, they are more ambiguous in Germany.

The latter result is coherent with the expert survey. Experts across different regions in the world expect a decline in investment in their own countries. Especially for Canada and Mexico, in emerging and advanced Asian economies, as well as major European economies with substantial US FDI, such as Germany and Ireland, experts expect a shift in investment toward the USA. In addition, negative perceptions (e.g., expecting investment to move to the US) are far more frequent in countries with moderately to high marginal effective tax rates (METR) that now exceed those of the USA (see Panel D of Table 5).^17^ All else being equal, those countries offered lower corporate taxes than the USA before the reform, but have now lost this advantage. Nevertheless, respondents from the USA are more skeptical about the effects of the tax reform (see Panel C of Table 5). Just over half of US respondents agree that investment will rise in the USA, and this while one of the main aims of the tax reform was to boost domestic investment in the USA.

### Trade

As a third empirical exploration, we examine survey responses regarding possible effects of the reform on exports to the USA and imports from the USA to Germany.^18^ Trade may be affected through changes in the location of economic activity in response to the reform and through tax deductions for exports within the scope of the FDII provision. While the effect on exports and imports is limited across the full sample (Table 6, Panel A), planned trade and investment responses are positively correlated. Among the firms with growing US investment, 11% intend to import more from the USA and 34% plan on increasing their exports to the USA, compared to 14% who intend to export less to the USA. The idea that firms may replace exports to the USA by products produced in the USA finds little support in our survey data. Along similar lines, among the firms cutting back on US investment, 70% intend to import less from the USA and 49% expect they will export less to the USA. Investment and trade seem to be complements, rather than substitutes. The second firm survey (Panel B) indicates only minor effects of the reform on US imports of German firms. However, a quarter of firms with US production expect to increase its US sales. Yet, it is not possible to distinguish whether these originate from US production or from exports from Germany to the USA. Also, rising sales might likewise be due to the US personal income tax provisions stimulating US demand.

**Table 6:** Effects on trade

	N	Decrease (%)	No change (%)	Increase (%)
*Panel A: First ifo firm survey March 2018*				
Imports from the US				
All firms	3430	4	95	1
Firms with at least 5% US revenue	490	5	93	1
By investment choice				
Firms planning to increase investment in the US	99	5	84	11
Firms planning to decrease investment in the US	147	70	30	0
Firms planning to increase investment in Germany	200	33	63	5
Firms planning to decrease investment in Germany	57	37	54	9
Exports to the US				
All firms	3483	3	95	2
Firms with at least 5% US revenue	528	4	89	8
By investment choice				
Firms planning to increase investment in the US	104	14	52	34
Firms planning to decrease investment in the US	145	49	49	2
Firms planning to increase investment in Germany	205	19	73	8
Firms planning to decrease investment in Germany	57	44	44	12

	*N*	All firms (%)	Firms with US production (%)	
*Panel B: Second ifo firm survey April/May 2018*				
Increased inputs from US	1286	1	7	
Increased sales in the US	186	4	25	

	*N*	Decrease (%)	No change (%)	Increase (%)
*Panel C: ifo expert survey April 2018*				
USA	42	33	48	19
EU15	270	27	61	11
Newer EU members	126	15	73	12
Other advanced economies	97	31	47	22
CIS and Emerging Europe	86	24	68	8
Emerging Asia	46	51	39	10
Latin America	105	39	47	14
Africa	102	22	62	16

This table outlines whether firms plan to export/import more or less intermediated or final products in reaction to the TCJA (Panels A and B). Panel C states the evaluation of experts of the impact of TCJA on the trade balance of their country

The expert survey also assessed if the tax reform influenced countries’ net exports. With the US president frequently criticizing the US trade deficit, trade effects also figure prominently in the political discussion. Overall, assessments are more ambiguous as shown in Panel C of Table 6. While 20% of US experts expect net exports to increase, a third expect a decrease, in line with 38% of experts in other countries around the world. Experts in Asian countries as well as Latin America, although to a lesser degree, are likely to envisage decreasing net exports. Experts in other advanced economies have the comparatively highest likelihood of expecting an increase.

### Effects on profit shifting and headquarter location

Besides having an impact on tax revenues, investment, and the balance of trade, the TCJA’s provisions affect profit shifting and encourage the location of intellectual property rights in and the repatriation of offshore profits to the USA.

Yet, virtually none of the firms in the first ifo business survey intend to adjust the location of their IP (not depicted here). At a first glance, this contrasts with the world expert survey: As Table 7 shows, roughly half of all US respondents expect the location of intellectual property rights to shift toward the USA. The discrepancy between both surveys may be due to the fact that IP susceptible to profit shifting would presumably be neither held in Germany nor in the USA, but rather in a tax haven.

**Table 7:** Survey results of the expert survey—effects of the tax reform

	*N*	Profit shifting to country			Location of IP rights			Relocation of headquarters			Repatriation of offshore profits		
		+	=	−	+	=	−	+	=	−	+	=	−
USA	42	66	29	5	53	48	0	58	43	0	80	20	0
EU15	269	11	58	31	4	77	19	7	72	22	10	62	28
Newer EU members	124	8	85	7	2	86	12	7	81	12	15	81	4
Other advanced economies	97	17	50	33	3	74	22	8	71	22	15	60	25
CIS and Emerging Europe	86	7	78	15	2	89	9	14	71	15	8	73	18
Emerging Asia	46	11	61	28	16	55	29	12	62	26	17	56	27
Latin America	105	14	62	24	9	76	15	11	71	18	16	69	15
Africa	102	13	76	11	20	75	5	5	89	6	8	84	8

Table shows the respective answers of the ifo expert survey regarding profit shifting, location of IP rights and headquarters as well as repatriation of offshore profits. “+,” “=” and “−,” denote “increase,” “no change” and “decrease,” respectively. The numbers represent percentages

Negative effects on the location of IP are predominantly feared in Asia and in advanced economies, including the EU-15, with the most negative assessment in Ireland, a country with strong incentives to hold IP, and Canada. Positive assessments occur more frequently in emerging economies. However, responses do not differ much between countries with and without an IP box regime.^19^ In addition to strategically locating IP rights, multinational companies have access to additional strategies, for example shifting profits to low-tax jurisdictions. 66% of US respondents expect that more profits will be shifted toward the USA following the reform. The picture varies between other countries: around 30% of experts in advanced economies, in- and outside the EU, as well as in Asian economies, expect that profits will be shifted away from their countries, while this is expected by fewer experts in other regions of the world. Here, it seems to make a difference whether a country has an IP box in place. While 31% of experts in countries with IP regimes expect decreasing profit shifting, this only applies to 20% in other countries. By contrast, around 12% of the respondents expect that more profits will be shifted toward their country.

In recent years, several large US companies raised substantial attention in the media as they relocated their legal residence to a low-tax country such as Ireland (Jolly 2016). On average, companies reduced their effective tax burden, measured by the ratio of worldwide tax payments to profits, from 29% to 18% via corporate inversions (Congressional Budget Office 2018). On the one hand, the US tax reform’s shift from a global toward a territorial tax system reduces incentives to invert, as US corporations are now only liable to pay taxes on their US profits. On the other hand, some relocation incentives remain. Some of the provisions of the Tax Cuts and Jobs Act specifically apply to US corporations, and corporate inversions could still be attractive to avoid GILTI taxes. Nevertheless, over half of US respondents believe the reform will result in an increasing number of headquarters being located in the USA. As before, countries located close to the USA like Canada, Mexico and some further Latin American countries, as well as those with substantial US FDI, such as Ireland, Switzerland, the UK and Germany, tend to expect the most negative impact. A similar finding applies to emerging Asian countries, with Chinese respondents more often expecting a relocation of headquarters than respondents in smaller Asian countries. By contrast, positive evaluations are not as concentrated across countries, but tend to occur more often in Emerging Europe, Asia and Latin America.

Prior to the reform, the deferral of taxation until the repatriation of profits leads to a substantial accumulation of profits in foreign subsidiaries, often located in low-tax jurisdictions. Moody’s estimated that US non-financial corporates’ offshore cash holdings amounted to $1.4 trillion in 2017 (Moody’s 2017). Including re-invested profits, the Joint Committee on Taxation estimated that undistributed offshore earnings and profits even amounted to $2.6 trillion in 2015 (Joint Committee on Taxation 2016; Keightley 2013). Under the TCJA, foreign profits are exempt from US tax. However, the transition tax charged on non-repatriated past foreign profits results in large one-time tax payments for many companies. As a result of these changes, around 80% of US respondents expect an increase in the repatriation of offshore profits to the USA. Across the world, decreasing foreign cash holdings are expected by 23% of all experts, while 14% expect offshore profits to rise in their country. Negative perceptions are particularly high in some countries. According to a Congressional Research Service Report (Keightley 2013), 43% of US corporations’ overseas profits were reported in Bermuda, Ireland, Luxemburg, the Netherlands and Switzerland. Unsurprisingly, experts in those countries anticipate a particularly large impact, with 43% predicting a decrease in reported earnings in their country. Among the remaining countries, experts in advanced economies and Asian countries tend to expect a negative outcome. Overall, negative anticipations are most frequent in countries with very low marginal effective tax rates, as well as countries with moderate tax rates that now exceed those of the USA.

## Conclusion

The Tax Cut and Jobs Act drastically altered the US tax system, also with substantial implications for multinationals with ties to the USA. We assess firm-level survey data from Germany as well as global survey data to gauge the reform’s impact around the world and provide insights into policymakers on firms’ responses to the reform.

Our results indicate that while the majority of German firms do not plan on adjusting investment, many firms with considerable US exposure respond to the reform: About a third of German firms that expect to benefit from the tax cut and/or that have capacities in the US intend to invest more in the USA. Effects on German domestic investment are more ambiguous: Some firms expand capacities in both locations, which may be driven by the TCJA’s stimulus to US demand, by firms’ increase in after-tax profitability, as well as by the TCJA’s GILTI provision incentivizing the increase of tangible assets in high-tax countries.

However, other firms intend to substitute between investment in both countries. This indicates that for some firms, the TCJA constitutes an impediment to Germany’s competitiveness in attracting multinational investment. However, while our results speak in favor of a substitution effect between US and German investment, our results do not indicate that many firms substitute US investment for German exports.

Overall, our results show that the reform triggers significant firm responses. When supplementing our analysis with worldwide survey data, our results also point to a negative impact on tax revenues and investment in other countries with close economic ties to the USA. Since the reform, tax competition has intensified, and further countries such as France and Belgium have lowered their corporate tax rates. This further worsens the competitive position of other high-tax countries, such as Germany, and may warrant tax reforms that incentivize domestic investment. When designing a tax reform, policymakers should not only think about rate cuts, but consider possible interaction effects with the TCJA’s international provisions.

## Footnotes

1 This paper is partially based on Krolage and Wohlrabe (2018), Rathje and Wohlrabe (2019) and Boumans and Krolage (2018).

2 Effects on debt-financed investments are not as clear-cut (Chalk et al. 2018; Gravelle and Marples 2020). In addition, the TCJA limits the deductibility of interest expenses, which reduces debt bias in investment choices.

3 See, e.g., https://www.brookings.edu/blog/up-front/2017/10/25/repatriated-earnings-wont-help-american-workers-but-taxing-those-earnings-can/.

4 These provisions are temporarily suspended under the Coronavirus Aid, Relief, and Economic Security Act.

5 See Sauer and Wohlrabe (2018) for further details on the construction of the index and Lehmann (2020) for a comprehensive survey of its forecasting properties.

6 The regular survey is constructed as a panel. New firm acquisitions are done using stratified random samples.

7 At times, the ifo Institute adds special questions which are related to policy issues such as the introduction of the minimum wage or potential consequences of the Brexit. The underlying microdata of regular and special questions have been used in various studies. See, among others, Bachmann et al. (2013), Strasser (2013), Huber (2018) or Enders et al. (2019).

8 This refers to question 3 and 4 in Appendix. The first question with respect to US revenue was answered by 5405 firms.

9 The strata were size, branch and legal form. The random sample was drawn from the Orbis database.

10 More details on the survey can be found in Stiftung Familienunternehmen (2018).

11 An economic expert and someone who is eligible for participating in the World Economic Survey is someone who works with economic data and has a good understanding of the economic developments within their own country.

12 The WES, compiled by the ifo Institute since 1981, aims at providing a timely and accurate picture of the current economic situation and economic trends over 100 key advanced, emerging and developing economies by polling more than 1000 experts quarterly. In selecting experts, emphasis is placed on their professional competence in economic matters and inside knowledge of their countries. See Boumans and Garnitz (2017) for further details. The survey has been proven to predict business cycles quite well, cf. Kudymowa et al. (2013) or Garnitz et al. (2019). More studies have used the supplementary question for further research, cf. Boumans et al. (2018).

13 80% of the respondents have a master or PhD degree and 70% studied economics. 43% of the respondents are affiliated with a university, research institution or think-thank and 15% work at banks or central banks.

14 This might be explained by the bilateral investment agreement between the Netherlands and the USA, the so-called Dutch-American Friendship Treaty (DAFT), which provides national treatment and free entry for foreign investors. Another explanation might be that although it is a large recipient of American FDI, the Netherlands is also a key export platform and pan-regional distribution hub for US firms (US Department of State 2017).

15 The US category consists of 36 respondents from the USA.

16 Becker and Riedel (2012) show that multinational firms benefitting from national tax cuts sometimes expand their activities in other countries as well.

17 Marginal effective tax rates are based on the analysis of Mintz (2018).

18 Note that while we explicitly asked for responses to the TCJA, the survey was conducted at a time of high uncertainty regarding US trade policy. Threats of tariffs between the USA and the EU might have hence impacted responses.

19 Countries are classified as having an IP regime based on the OECD’s assessment in OECD (2017).
